# The Impact of Reconstruction Methods, Phylogenetic Uncertainty and Branch Lengths on Inference of Chromosome Number Evolution in American Daisies (*Melampodium*, Asteraceae)

**DOI:** 10.1371/journal.pone.0162299

**Published:** 2016-09-09

**Authors:** Jamie McCann, Gerald M. Schneeweiss, Tod F. Stuessy, Jose L. Villaseñor, Hanna Weiss-Schneeweiss

**Affiliations:** 1 Department of Botany and Biodiversity Research, University of Vienna, Rennweg 14, 1030 Vienna, Austria; 2 Herbarium, Department of Evolution, Ecology, and Organismal Biology, Ohio State University, 318 W. 12th Ave., 43210 Columbus, Ohio, United States of America; 3 Instituto de Biologia, Departamento de Botánica, Universidad Nacional Autónoma de México, Tercer Circuito s/n Ciudad Universitaria Delegación Coyoacán Apartado Postal 70-233, 04510 México, D.F., México; Institute of Botany, CHINA

## Abstract

Chromosome number change (polyploidy and dysploidy) plays an important role in plant diversification and speciation. Investigating chromosome number evolution commonly entails ancestral state reconstruction performed within a phylogenetic framework, which is, however, prone to uncertainty, whose effects on evolutionary inferences are insufficiently understood. Using the chromosomally diverse plant genus *Melampodium* (Asteraceae) as model group, we assess the impact of reconstruction method (maximum parsimony, maximum likelihood, Bayesian methods), branch length model (phylograms versus chronograms) and phylogenetic uncertainty (topological and branch length uncertainty) on the inference of chromosome number evolution. We also address the suitability of the maximum clade credibility (MCC) tree as single representative topology for chromosome number reconstruction. Each of the listed factors causes considerable incongruence among chromosome number reconstructions. Discrepancies between inferences on the MCC tree from those made by integrating over a set of trees are moderate for ancestral chromosome numbers, but severe for the difference of chromosome gains and losses, a measure of the directionality of dysploidy. Therefore, reliance on single trees, such as the MCC tree, is strongly discouraged and model averaging, taking both phylogenetic and model uncertainty into account, is recommended. For studying chromosome number evolution, dedicated models implemented in the program ChromEvol and ordered maximum parsimony may be most appropriate. Chromosome number evolution in *Melampodium* follows a pattern of bidirectional dysploidy (starting from *x* = 11 to *x* = 9 and *x* = 14, respectively) with no prevailing direction.

## Introduction

Chromosome number change plays an important role in eukaryotic evolution in general and in plant diversification and speciation in particular [1, 2]. Several types of chromosome number change are commonly considered. Dysploidy is the homoploid change of the chromosome base number via chromosomal rearrangements without significant loss of genetic material [3]. Its evolutionary significance is evident from a high diversity of chromosome numbers even among closely related groups [1, 4, 5], where the distribution of chromosome base numbers often correlates with phylogenetic relationships [6–8]. Polyploidy is the multiplication of entire chromosome sets. It has become a major focus in plant evolutionary biology due to the recognition of the ubiquity of polyploidy in angiosperms via identification of several rounds of whole genome duplication affecting even small angiosperm genomes [9, 10]. Auto- and especially allopolyploidy are important drivers of diversification, both in speciation and as a trigger for genomic and genetic changes (reviewed in [11]), such as in many evolutionarily young plant crops [12]. Aneuploidy refers to the loss or gain of entire chromosomes and thus of genetic material, which is rarely tolerated by plants [3]. Like the presence of accessory chromosomes (B-chromosomes), aneuploidy is usually transitory and hence plays only a minor role in evolutionary terms [1].

Prerequisites for a solid analysis of chromosome number change are comprehensive and unambiguous chromosome number data and sound hypotheses on phylogenetic relationships [8, 13–15]. Over the last decades, enormous progress has been made on both aspects. Chromosome numbers are known for a fair number of plants (although with conspicuous gaps in, for instance, tropical lineages), many of them available in the Chromosome Counts Database [16]. Likewise, phylogenetic hypotheses, established by application of increasingly sophisticated phylogenetic methods with an increasing amount of data, are available for many plant groups [17].

Chromosome number evolution (and ancestral character state reconstruction in general) can be inferred using a number of formal approaches. A commonly used method is maximum parsimony (e.g., [18, 19]). Whereas unordered parsimony makes no assumptions about state transitions, ordered parsimony allows only transitions between consecutive chromosome numbers, thus implicitly accounting for unobserved intermediate character states. Based on mechanisms of chromosome number change [20], the assumption of ordered states appears to be more realistic. Among the disadvantages of maximum parsimony are multiple transitions on a single branch (beyond those implicated by ordered parsimony) that are not accounted for [21] (if changes in chromosome number are connected to speciation, this may, however, be of limited concern) and that statistical comparison of different reconstruction schemes is not possible [22].

Alternative methods are model-based and employ explicit probabilistic models to describe how character evolution has proceeded [21]. By taking branch lengths into account, these models allow for unobserved state changes, i.e., multiple transitions. This may, however, result in substantial differences in inferences from phylograms, where branch lengths are proportional to molecular evolution, from those on ultrametric trees (chronograms), where branch lengths are proportional to (absolute or relative) time despite identical topologies [23]. Although it may be argued that the only meaningful interpretation of these models is that the probability of change depends on time, measuring branch lengths in units of “opportunity for selection”, such as genetic distances [24], should not be disregarded a priori [23]. An advantage of model-based methods over parsimony is that the fit of different models can be readily compared using statistical model comparison approaches, such as applying information criteria or using Bayes Factors [25].

For character state reconstruction, most commonly a continuous-time Markov model is used, which contains maximally *n* (*n* − 1) rate parameters, *n* being the number of character states, describing the transition between character states [26, 27]; this model is available in both maximum likelihood and Bayesian implementations. As this model does not account for character states unobserved among included samples, its applicability in the context of chromosome number evolution may be compromised. Mayrose et al. [28] introduced a set of continuous-time Markov models, whose parameterization is tailored for the study of chromosome number evolution. The basic model parameters are the rate of gain and the rate of loss of a single chromosome (thus, unobserved chromosome numbers are accounted for) and the rate of chromosome number duplications. Additional models of dysploid change can be implemented by incorporating parameters introducing a linear dependency of chromosome gain and loss from the current chromosome number; additional models of polyploid change can be implemented by incorporating a rate of chromosome number demi-duplication (i.e., a 1.5 fold increase of the chromosome number) and/or a rate of chromosome multiplication in general (controlled by a rate parameter and a parameter describing the monoploid base number: [29]). Single parameter models (e.g., one including a single rate of dysploid change) are conceivable, but have not been implemented [28, 29]. For the models of Mayrose et al. [28], no Bayesian implementation allowing to set priors on the model parameters is available.

Irrespective of reconstruction method used, inferences will be affected by phylogenetic uncertainty, i.e., uncertainty with respect to topology and/or branch lengths (the latter not relevant for parsimony reconstruction). Although this can be accounted for by performing character state reconstruction over a set of trees (e.g., the posterior set of trees from a Bayesian analysis; a bias for overestimating transitions may, however, remain: [30]), reconstructions often use only a single representative topology, such as the majority-rule consensus tree (e.g., [31]) or the maximum clade credibility (MCC) tree (e.g., [32, 33]). It remains, however, unclear how representative these single trees are.

A well suited system to study chromosome number evolution is the genus *Melampodium* (Asteraceae). It comprises 40 species centered in Mexico and Central America, a few reaching the southwestern United States and South America [34]. Chromosome numbers are known for all species except one [35]. Apart from 13 exclusively polyploid species, mostly of allopolyploid origin [34–36], the remaining species are exclusively (23 species) or mostly diploid (four species with both diploid and tetraploid cytotypes [35]). In diploids, five chromosome base numbers are found (*x* = 9, 10, 11, 12, 14: [35]), whose distribution largely corresponds with the delimitation of morphologically and phylogenetically defined sections [34, 37]. Previous intuitive analyses suggested *x* = 10 or *x* = 11 as the ancestral chromosome base number [35, 38, 39], but both hypotheses may be flawed, because the presumed outgroups used for character state polarization, *Acanthospermum* and *Lecocarpus*, have recently been shown to be nested within *Melampodium* [37].

Here, we assess the impact of reconstruction method, branch length model (phylograms versus chronograms) and phylogenetic uncertainty on the inference of chromosome number evolution using *Melampodium* as model group. To this end, we use ordered parsimony, maximum likelihood (using both standard Markov models for discrete multistate characters and the models devised by Mayrose et al. [28]), and a Bayesian method on posterior sets of both phylograms and chronograms. Although more decisive results on the performance of methods can be achieved using simulations, we consider the analysis of empirical data using a set of appropriate methods a valuable complementary approach, as only empirical data sets are guaranteed to represent realistic settings. By reconstructing ancestral chromosome numbers and estimating rates of chromosome number change, previous hypotheses concerning chromosome base numbers (*x* = 10 versus *x* = 11) in *Melampodium* and the directionality of dysploidy (ascending, i.e., with increasing chromosome base number; descending, i.e., with decreasing chromosome base number; or bidirectional) can be tested.

## Materials and Methods

### Phylogenetic analysis

Sequences of the nuclear rDNA region, comprising the 3'-end of the 18S-gene, the Internal Transcribed Spacer 1, the 5.8S gene, the Internal Transcribed Spacer 2 and the 5'-end of the 26S gene and henceforth jointly referred to as ITS, and the plastid *trnK*-intron including the *matK*-gene, henceforth referred to as matK, were obtained from Blöch et al. [37] (S1 Table). These data sets include all species of *Melampodium*, half (ITS) to one sixth (matK) of *Acanthospermum* species and half (ITS) to all (matK) of *Lecocarpus* species. These datasets were trimmed to include only diploid accessions because most of the polyploids in *Melampodium* are either of allopolyploid origin [36] or of likely recent origin (in species with both diploids and polyploids: [35]) with accordingly higher polyploidization rates resulting in polyploidization rate heterogeneity across time, which may bias inference of phylogeny-wide polyploidization rates. Testing any bias, which, as suggested by a reviewer, may actually result from this very exclusion of recent polyploids, would require simulations going beyond the scope of this study. Even if a bias exists, this should not affect the comparison of different reconstruction methods, as these use the same data sets. *Melampodium moctezumum* was excluded from the analyses, because its exact chromosome number is not known. Although classified as separate genera [34], *Acanthospermum* and *Lecocarpus* are phylogenetically nested in *Melampodium* [37] and were, therefore, included in the analyses. Each species and each intraspecific taxon (varieties in *M*. *cinereum* and *M*. *montanum*) was represented by a single accession except in cases of intraspecific sequence variation exceeding an *ad hoc* threshold. Briefly, inter- and intraspecific pairwise distances were calculated using K2P distances with MEGA 4 [40] and the distance threshold was defined as the median value of interspecific distances in the distance range, where inter- and intraspecific distances overlapped. The median value was preferred over alternative cut-offs, such as the mean or the minimum interspecific distance, as it avoids unduly strong influence of very small interspecific distances. Intraspecific sequence data whose pairwise distances exceeded this threshold were kept in the dataset. The final datasets comprised 39 accessions in the ITS dataset and 34 accessions in the matK dataset (S1 Table).

Due to highly supported incongruences between nuclear and plastid phylogenies [37] data sets were analyzed separately. The best fit substitution models were identified using MODELTEST 3.6 [41]. For ITS, the dataset was divided into the rDNA partition (partial 18S and 26S genes, complete 5.8S gene) and the combined spacers (ITS 1 and 2) partition. For the former there was a high uncertainty concerning the best fit model (20 models until the cumulative Akaike weight exceeded 0.95) ranging from two to nine free parameters; eventually a moderately complex model was chosen (HKY+Γ with 5 free parameters), incorporating invariable sites, often parameterized separately as proportion of invariable sites I, in the gamma distribution (due to identifiability issues: [42]) modeled with six discrete rate categories. For the spacers partition, only three models were included with eight to ten free parameters until the cumulative Akaike weight exceeded 0.95, and a GTR+Γ model was used. Although model uncertainty was higher for the *trnK*-intron partition than for the *matK*-partition of matK (nine models with six to nine parameters versus four models with eight to ten parameters, respectively, until the cumulative Akaike weight exceeded 0.95), for both partitions the GTR+Γ model was selected. Phylograms, i.e., trees where branch lengths are proportional to the number of evolutionary events (here substitutions per site), were constructed using MrBayes 3.1.2 [43]. This version of MrBayes uses branch-length priors that may result in an overestimation of tree-lengths, but this should not significantly affect relative branch lengths [44]. We employed three runs with four chains each (three heated ones using a heating parameter of 0.1) for 25 × 10^6^ generations sampling every 15,000^th^ generation. The first 10% were discarded as burn-in, which was well after the chains had reached stationarity (standard deviations of split frequencies being below 0.01 and ESS values being safely above 1,000), and a final set of 4,500 trees was used for all further analyses. Trees were rooted using *Galinsoga* (*x* = 8, 9: [45]) and *Milleria* (*x* = 15: [46]), but these outgroups were pruned from the trees prior to ancestral chromosome number reconstruction analyses. Chronograms, i.e., trees where branch lengths are proportional to (absolute or relative) time, were constructed using BEAST 1.4.x [47] with a speciation model following a Yule process as tree prior and separate relaxed clocks for each data partition with calibrations achieved via normal priors on each partition’s substitution rate (given as mean/standard deviation): rDNA 0.0002/0.0002, based on rate estimates by Kuzoff et al. [48] for 18S and 26S genes relative to plastid *rbcL* genes, using *rbcL* substitution rates for asterids from Bremer and Gustafsson [49]; ITS spacer 0.005/0.0025, based on ITS substitution rates for herbaceous plants summarized by Kay et al. [50]; *trnK*-intron 0.004/0.002 and *matK* 0.0022/0.0011, both based on rate estimates given by Yamane et al. [51]. As our interest here was not in molecular dating, we neither conducted testing with respect to the used clock models nor fine-tuned the calibration priors. As the coefficient of rate variation for the rDNA partition of ITS abutted zero (data not shown), we conducted additional likelihood analyses using a strict clock model for the rDNA partition; these analyses yielded nearly identical results with respect to chromosome number reconstructions and the used test statistics (data not shown) and, hence, were not pursued any further. For each data set, three runs for 50 × 10^6^ generations sampling every 30,000^th^ generation were employed; again, the first 10% were discarded as burn-in (ESS values being safely above 1,000), and a final set of 4,500 trees was used for all further analyses. Sequence alignments and phylogenetic trees are available from the Dryad Digital Repository at http://dx.doi.org/10.5061/dryad.6r12h.

### Ancestral chromosome base number reconstructions

For the following analyses, haploid chromosome numbers (*n*) were used. All analyses were performed on each of the four 4,500-tree data sets (i.e, ITS and matK with MrBayes and BEAST, henceforth termed ITS-MB, matK-MB, ITS-B and matK-B). Prior to analyses, these trees were rescaled to an equal length of five, i.e., the number of different character states (the default scaling in ChromEvol 2.0).

#### Test statistics

For comparison of reconstruction uncertainty resulting from differences in reconstruction method we used the proportion of the most frequently reconstructed chromosome number and normalized it giving an index of reconstruction precision per node *n*, *RP*_*n*_, of

$$\begin{array}{c} RP_{n}=\frac{max_{j\in X}\left(P_{j}\right)-P_{min}}{P_{max}-P_{min}}, \end{array}$$ (1)

where *P*_*j*_ is the reconstruction proportion of character state *j* and *X* is the set of all character states reconstructed with probability > 0 (or a user-defined cutoff, here 10^−2^) at *any* node of the tree (or of a set of compared trees), i.e., *X* = *X*_1_ ∪ *X*_2_ ∪ … ∪ *X*_*N*_, where *N* is the number of nodes with reconstructions in the tree (or of a set of compared trees). *P*_*max*_, the maximum possible proportion of the most frequently reconstructed character state, is 1; *P*_*min*_, the minimum possible proportion of the most frequently reconstructed character state, is 1/*C*_*X*_, where *C*_*X*_ is the number of character states in *X*. Therefore, *RP*_*n*_ can be reformulated as

$$\begin{array}{c} RP_{n}=\frac{C_{X}\cdot max_{j\in X}\left(P_{j}\right)-1}{C_{X}-1}. \end{array}$$ (2)

The normalization is necessary, as different nodes of the same tree (or of a set of compared trees) can have different sets of reconstructed character states, because states may have (nearly) zero probability at some nodes, but not at others. For example, for a node with chromosome number reconstruction probabilities of 0.5, 0.5, 0 and 0, *RP*_*n*_ will be 4 × 0.5 − 1/(4 − 1) = 0.33.

Reconstruction precision for an entire tree, *RP*_*t*_, was defined as the arithmetic mean of the tree’s *RP*_*n*_ values. These indices of reconstruction uncertainty ranged from 0 (minimum precision and maximum uncertainty) to 1 (maximum precision and no uncertainty). Here, we focused on the *RP*_*t*_ value of the 95% majority rule consensus trees and on the range of *RP*_*n*_s on the consensus trees.

For quantifying directionality of dysploidy, we used the difference between the number of chromosome gains and number of chromosome losses, henceforth abbreviated as G-L. Thus, positive values indicate prevalence of gains and negative values indicate prevalence of losses for a particular tree and analytical method.

#### Maximum Parsimony (MP)

The parsimony algorithm was implemented using the program Mesquite 2.75 [52]. Chromosome number was coded as an ordered multistate character. The inferred state(s) for the nodes in each tree were printed to a single results file using a script (available from http://mesquiteproject.wikispaces.com/Scripts+%26+Macros). This results file was parsed and ancestral states for each node were mapped onto the tree using custom python scripts and the DendroPy Phylogenetic Computing Library 3.12.0 [53]. The numbers of gains and losses were calculated by traversing the tree from root to leaves and summing up the difference between the parent and child nodes. This method included ambiguous reconstructions by taking into account all possible transitions for nodes with more than one state.

#### Maximum Likelihood (ML)

Reconstructions using model-based approaches and the likelihood criterion were performed in two different programs. The first analysis was performed in ChromEvol 2.0 [29], henceforth referred to as ML-CE. This program was developed specifically to investigate chromosome base number evolution with a number of models available that include dysploidy (with a constant or a linear rate) without or with polyploidy and, in the latter case, without or with demi-polyploidy, resulting in a total of eight models. Model fit of each of these models was assessed using the Akaike Information Criterion (AIC).

The second analysis using maximum likelihood was performed in BayesTraits 2.0 [27], henceforth referred to as ML-BT. Its implementation allows for the specification of arbitrarily complex models of evolution and is generally applicable for reconstructing the evolution of any discrete character. For the same reasons given for ordered parsimony, changes were allowed to occur only between neighboring states (i.e., 9 ↔ 10 ↔ 11 ↔ 12 ↔ 14). Three models were analyzed differing in the number of rate parameters. The first allowed only one rate class (*one-rate*) where all rates were the same, while the second allowed forward and reverse rates to differ (*two-rate*) and the third allowed all changes to have a unique rate (*multi-rate*). Model fit was accessed using the AIC.

Chromosome number reconstructions taking model uncertainty into account were obtained via model averaging using Akaike weights [54]. Specifically, the model averaged chromosome number probability, $\hat{\bar{CN}}$, was calculated as the weighted arithmetic mean of the chromosome number probabilities using the Akaike weight of the reconstruction model they were obtained from.

#### Bayesian Analysis (BI)

Bayesian reconstructions were done using BayesTraits 2.0, employing the same rate models as used for the BayesTraits maximum-likelihood analysis described above. Prior distributions for the rate parameters were modeled via gamma distributions, whose mean and variance were described by hyperpriors with a uniform distribution bound between 0 and 1, thus safely including the empirical Bayes estimates of mean and variance derived from plotting the rates from the maximum-likelihood analysis over all 4,500 trees (data not shown). The MCMC chain was run for 4.6 × 10^7^ generations with an initial burn-in of 10^6^, which is well after the chains had reached stationarity (ESS values being safely above 1,000 with the exception of the *multi-rate* model analysis of ITS-MB, where ESS values ranged from 127 to nearly 400). This same procedure was performed using each of the three rate models described above for the ML-BT analysis.

Model testing was performed using Bayes Factors. Marginal log likelihoods were approximated via harmonic means of the log-likelihood as calculated by BayesTraits. As test statistic *logBF* = 2 × (*log*[*HM*(*model*1)] − *log*[*HM*(*model*2)]) was used, with *logBF* > 2 indicating positive evidence for model 1 [55]. We acknowledge that better methods for estimating marginal likelihoods are available [56], but none of these are implemented in BayesTraits.

Chromosome number reconstructions taking model uncertainty into account were obtained via the reversible jump MCMC implemented in BayesTraits [57], henceforth referred to as BI-RJ. This approach allows searching the posterior distribution of models differing in the number and assignments of rate classes as well as the posterior distributions of their parameters. To permit sufficient, yet not exhaustive exploration of model space (there are more than 51 trillion models for 5 character states and thus 20 rates), the analysis was run for 451 × 10^6^ generations, removing the first 10^6^ generations as burn-in (ESS values above 10,000) and sampling every 500^th^ generation. Prior distributions for the rate parameters were modeled via gamma distributions, whose mean and variance were described by hyperpriors with a uniform distribution bound between 0 and 1.

## Results

### Phylogenetic resolution

**Fig 1:**
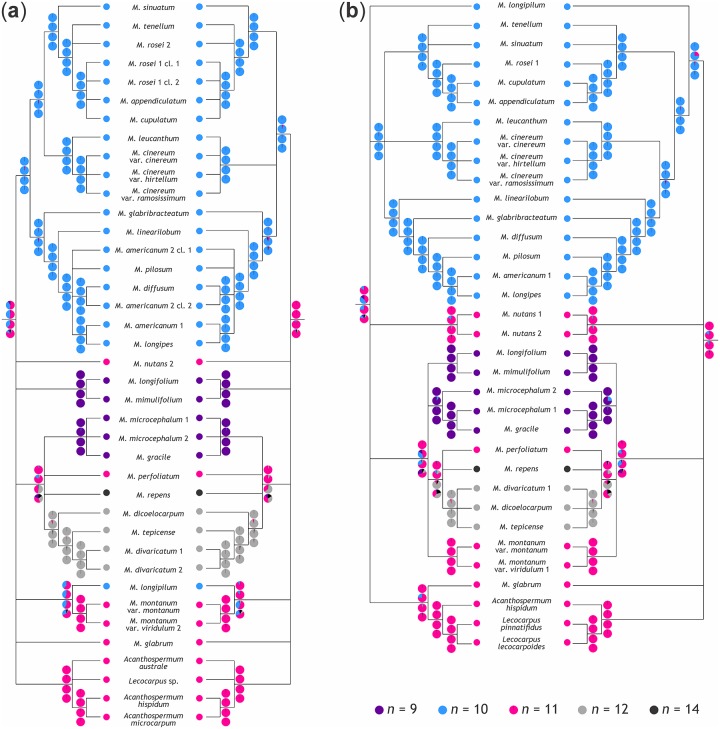
Chromosome number reconstructions in *Melampodium*. Chromosome number reconstructions plotted on 95% majority rule-consensus trees from phylogenetic analysis of (A) nuclear sequence data using BEAST (ITS-B, left) and using MrBayes (ITS-MB, right) and of (B) plastid sequence data using BEAST (matK-B, left) and using MrBayes (matK-MB, right). At each node, the average and, in case of maximum likelihood reconstructions, model-weighted probabilities of ancestral chromosome base numbers are shown (from top to bottom): ordered maximum parsimony (MP), maximum likelihood using ChromEvol (ML-CE), maximum likelihood using BayesTraits (ML-BT), Bayesian Inference using Reversible Jump (BI-RJ). The pie charts represent the fraction of probability that is associated with a particular chromosome number.

For ITS-B and ITS-MB, 21 and 23 nodes had posterior probabilities of at least 0.95 (Fig 1); as a fully resolved ITS tree has 38 nodes, 55 and 61% of nodes were well-supported. Of the remaining nodes (i.e., those collapsed to polytomies in Fig 1), seven and eight, respectively, were within clades with identical chromosome number. For both matK-B and matK-MB, 23 out of 33 nodes (i.e., 70%) had posterior probabilities of at least 0.95 (Fig 1). Of the remaining nodes, two each were within clades with identical chromosome number. This suggests that phylogenetic signal is sufficient to render analyses of chromosome number evolution meaningful.

### Model uncertainty

Of the eight models tested in ChromEvol (ML-CE), those including dependency of the rates of dysploid change on current chromosome number were never chosen as best model (ΔAIC to the best model 1.1100–16.2286; S2 Table). Whereas model uncertainty was negligible for the matK data set (the CRND model, which has a constant rate of dysploid change and no duplications, was supported in at least 98.8% of cases), it was more pronounced for the ITS data set, where the CRND model was chosen in 88.9% (ITS-B) or 60.8% (ITS-MB) of cases, respectively (Table 1). The second most-often best model (CRDD: compared to the CRND model it additionally includes demi-duplications with the same rate as duplications) accounted for 10.6% (ITS-B) or 39.2% (ITS-MB) of cases, respectively; it is noteworthy that in about two thirds of the cases, where CRDD was supported as the best model, CRND was not the second-best model (Table 1). Model uncertainty was considerable: average Akaike weights of the best model ranged from 0.3742 (ITS-MB) to 0.4643 (matK-B) and the average number of least supported models, i.e., those left once the cumulative Akaike weight had reached or exceeded 0.95, ranged from 1.4 to 2.2 (Table 2). The same was true for model selection uncertainty (i.e., the confidence in the selected model compared to the other candidate models): average ratios of Akaike weights of the second best and the best model ranged from 0.3792 (matK-MB) to 0.5947 (ITS-MB; Table 2).

**Table 1:** Minimum and maximum ΔAICs for the Constant Rate—No Duplication (CRND) model against other ChromEvol models and, in parentheses, the proportion of cases where they are better than the CRND model.

Dataset	Models						
	CRD	CRDD	CRDE	LRND	LRD	LRDD	LRDE
ITS-B	-0.954	-8.609	-7.979	1.038	3.038	-4.045	-3.571
	4.543	4.189	5.953	9.094	11.094	11.0943	13.517
	(0.02)	(10.82)	(7.24)			(0.27)	(0.20)
ITS-MB	1.943	-6.863	-6.234	2.667	4.667	-3.301	-2.648
	2.001	2.001	4.521	6.010	7.399	8.010	14.544
		(39.24)	(28.04)			(1.42)	(0.36)
matK-B	-1.733	-8.274	-7.646	0.344	2.344	-4.368	-3.125
	5.682	6.570	7.957	10.158	13.980	13.980	13.611
	(0.47)	(1.11)	(0.64)			(0.18)	(0.18)
matK-MB	-0.584	-7.861	-7.239	0.890	2.890	-4.189	-3.569
	4.543	4.662	7.508	7.705	9.862	9.466	11.751
	(0.18)	(0.84)	(0.60)			(0.27)	(0.20)

Each data set (ITS-B—nuclear sequence data analyzed using BEAST; ITS-MB—nuclear sequence data set analyzed using MrBayes; matK-B—plastid sequence data analyzed using BEAST; matK-MB—plastid sequence data analyzed using MrBayes) has been analyzed under each of eight models implemented in ChromEvol 2. The predominantly supported model (CRND—Constant Rate—No Duplication model) has been compared against the remaining models (CRD—Constant Rate—Duplication only; CRDD—Constant Rate—identical Demi-duplication and Duplication; CRDE—Constant Rate—Demi-duplication Estimated; LRND—Linear Rate—No Duplication; LRD—Linear Rate—Duplication only; LRDD—Linear Rate—identical Demi-duplication and Duplication; LRDE—Linear Rate—Demi-duplication Estimated; see text for details).

**Table 2:** Model uncertainty and model selection uncertainty in maximum likelihood analyses using ChromEvol (values are given as averages and, in parentheses, ranges).

Dataset	Akaike Weight		Number of Excluded Models
	Best Model	2^nd^ Best / Best Model	
ITS-B	0.447 (0.245–0.737)	0.434 (0.168–1.000)	2.195 (1–6)
ITS-MB	0.374 (0.266–0.493)	0.595 (0.368–1.000)	1.400 (1–4)
matK-B	0.464 (0.234–0.699)	0.388 (0.130–1.000)	2.171 (1–5)
matK-MB	0.454 (0.238–0.650)	0.379 (0.140–1.000)	2.112 (1–4)

For each data set (abbreviations as in Table 1) model uncertainty has been quantified using the best model’s Akaike weight (ranging from 0 to 1: the higher the weight the lower model uncertainty; column “Best Model”); model selection uncertainty has been quantified using the ratio of Akaike weights from the second best against the best model (ranging from 0 to 1: the higher the value the higher model selection uncertainty; column “2nd Best / Best Model”).

Of the three models tested in BayesTraits (ML-BT), the *multi-rate* model was never chosen as best model (ΔAIC to the best model 4.3082–12.8234; S3 Table). Model uncertainty was moderate to low for all data sets, and the *one-rate* model was supported in 90.1% (ITS-MB) to 96.8% (matK-B) of cases (Table 3). The second most-often best model was the *two-rate* model; in about one fifth of the cases, where the *two-rate* model was supported as the best model, the *one-rate* model was the least supported (Table 3). Average Akaike weights of the best model ranged from 0.6074 (ITS-MB) to 0.6735 (matK-B) and average Akaike weights of the second best model, expressed as proportion of the Akaike weights from the best model, were from 0.4851 (matK-MB) to 0.6480 (ITS-MB); the average number of least supported models, i.e., those left once the cumulative Akaike weight had reached or exceeded 0.95, was 1 (Table 4).

**Table 3:** Minimum and maximum ΔAICs for the *one-rate* model against other BayesTraits models and, in parentheses, the proportion of cases where they are better than the *one-rate* model.

Dataset	Models	
	*Mult-Rate* vs. *One-Rate*	*Two-Rate* vs. *One-Rate*
ITS-B	-61.048	-69.774
	12.442	2.000
	(1.11)	(4.82)
ITS-MB	-60.881	-70.982
	10.498	1.597
	(1.80)	(9.87)
matK-B	-33.241	-43.139
	12.823	2.000
	(0.44)	(3.20)
matK-MB	-29.479	-37.926
	12.148	2.000
	(1.09)	(6.36)

Each data set (abbreviations as in Table 1) has been analyzed under each of three models implemented in BayesTraits 2. The predominantly supported model (*one-rate* model) has been compared against the remaining models (*two-rate* model, *multi-rate* model; see text for details).

**Table 4:** Model uncertainty and model selection uncertainty in maximum likelihood analyses using BayesTraits (values are given as averages and, in parentheses, ranges).

Dataset	Akaike Weight		Number of Excluded Models
	Best Model	2^nd^ Best / Best Model	
ITS-B	0.662 (0.500–1.000)	0.434 (0.002–0.992)	1.014 (1–2)
ITS-MB	0.607 (0.481–0.995)	0.648 (0.005–1.000)	1.017 (0–2)
matK-B	0.674 (0.499–0.993)	0.485 (0.006–0.995)	1.008 (1–2)
matK-MB	0.650 (0.496–0.996)	0.539 (0.004–1.000)	1.013 (1–2)

For each data set (abbreviations as in Table 1) model uncertainty has been quantified using the best model’s Akaike weight (ranging from 0 to 1: the higher the weight the lower model uncertainty; column “Best Model”); model selection uncertainty has been quantified using the ratio of Akaike weights from the second best against the best model (ranging from 0 to 1: the higher the value the higher model selection uncertainty; column “2nd Best / Best Model”).

In Bayesian analysis (BI), for all data sets Bayes Factors favored the *one-rate* model over the *two-rate* model (*2logBF* of 0.9253 in ITS-B to 1.4190 in matK-B) and over the *multi-rate* model (*2logBF* of 2.1137 in ITS-MB to 3.0787 in ITS-B; S4 Table). In the reversible-jump MCMC (BI-RJ; S5 Table), the average number of rate classes with non-zero rates was very similar among data sets (around 2.7). Likewise, for each data set the number of rates being zero fluctuated considerably (from 0 to 16) with its average ranging from 5.16 to 5.86. Although the number of times a particular rate was set to zero differed by an order of magnitude between the rarest and the most frequent one, only in a single data set, matK-MB, two rates (those pertaining to changes from *n* = 11 to *n* = 9 and from *n* = 11 to *n* = 10) were set to zero less than 5% of times.

### Reconstruction uncertainty

The average and, in case of maximum likelihood reconstructions (ML-CE, ML-BT), model-weighted probabilities of ancestral chromosome numbers from each analysis are shown on 95% majority rule consensus trees, where the pie charts represent the fraction of probability that is associated with a particular chromosome number (Fig 1). As we only consider clades with posterior probability of 0.95 or more, the effect of clades lacking in a subset of the posterior trees on the calculation of these probabilities is negligible.

Reconstruction precision statistics (*RP*_*t*_, minimum and maximum *RP*_*n*_) are provided in Table 5. Lack of resolution at the backbone (Fig 1) biases reconstruction precision statistics upwards, because information for basal nodes that are expected to have higher reconstruction uncertainty is lacking. However, as all reconstruction methods use the same set of trees, this systematic bias should affect all methods equally. Tree-wide reconstruction precision was highest (and reconstruction uncertainty was lowest) in MP reconstructions with *RP*_*t*_s ranging from 0.978 (ITS-B) to 1.0 (matK-MB). Likewise, node-related reconstruction uncertainty was usually lowest and varied the least with *RP*_*n*_s ranging from 0.741 (ITS-B) to 1.0 (all data sets). In contrast, tree-wide reconstruction uncertainty was highest in ML-BT with *RP*_*t*_s ranging from 0.926 (ITS-B) to 0.948 (matK-B); also node-related reconstruction uncertainty was highest and varied the most with *RP*_*n*_s ranging from 0.413 (ITS-MB) to 1.0 (matK-B). The other two methods, ML-CE and BI-RJ, had intermediate levels of reconstruction uncertainty. Whereas ML-CE outperformed BI-RJ with respect to tree-wide reconstruction uncertainty in the ITS data sets (*RP*_*t*_ scores of 0.970 and 0.980 versus 0.949 and 0.937 in ITS-B and ITS-MB, respectively), the reverse was true for the matK data sets (*RP*_*t*_ scores of 0.949 and 0.951 versus 0.954 and 0.967 in matK-B and matK-MB, respectively); ML-CE always outperformed BI-RJ with respect to both magnitude and variation of node-wise reconstruction uncertainty (*RP*_*n*_ range 0.713–1.0 versus 0.489–1.0). There was no clear relationship between reconstruction uncertainty and branch-length model (Table 5).

**Table 5:** Tree-wide and node-wise reconstruction precision (*RP*_*t*_ and *RP*_*n*_).

Dataset	Method	Reconstruction Precision		
		*RP*_*t*_ MRC	*RP*_*t*_ MCC	*RP*_*n*_ Range
ITS-B	MP	0.978	0.946	0.741–1.000
	ML-CE	0.970	0.984	0.814–1.000
	ML-BT	0.926	0.925	0.500–0.999
	BI-RJ	0.949	0.954	0.574–1.000
ITS-MB	MP	0.992	1.000	0.898–1.000
	ML-CE	0.980	0.992	0.871–1.000
	ML-BT	0.932	0.928	0.413–1.000
	BI-RJ	0.937	0.962	0.489–1.000
matK-B	MP	0.996	1.000	0.898–1.000
	ML-CE	0.949	0.983	0.720–1.000
	ML-BT	0.948	0.956	0.588–1.000
	BI-RJ	0.954	0.960	0.612–1.000
matK-MB	MP	1.000	1.000	0.993–1.000
	ML-CE	0.951	0.979	0.713–1.000
	ML-BT	0.947	0.971	0.578–1.000
	BI-RJ	0.967	0.978	0.623–1.000

Each data set (abbreviations as in Table 1) has been analyzed using each of four methods (MP—ordered Maximum Parsimony; ML-CE—Maximum Likelihood using ChromEvol; ML-BT—Maximum Likelihood using BayesTraits; BI-RJ—Bayesian Inference using Reversible Jump). Tree-wide reconstruction precision (*RP*_*t*_) has been calculated on the majority rule consensus tree (MRC) and on the maximum clade credibility tree (MCC); node-wise reconstruction precision (*RP*_*n*_) has been calculated for each of the posterior trees and is given as ranges.

Reconstruction uncertainty integrated over a set of trees can be high due to ambiguous reconstructions in the input trees (resulting in small *RP*_*n*_s in each tree) or due to unambiguous but contradicting reconstructions in the input trees (resulting in *RP*_*n*_s close to 1 in each tree). In the first case a tight correlation between integrated reconstruction uncertainty (shown on the nodes of the consensus tree; Fig 1) and individual reconstruction uncertainty (expressed as the proportion of input trees, where *RP*_*n*_ is at or above a certain threshold) is expected. Indeed, such a correlation was observed (Pearson’s correlation coefficient, *r*, ranging from 0.847 to 1) irrespective of the *RP*_*n*_ threshold (0.90 or 0.95) used (S6 Table).

### G-L distributions

**Fig 2:**
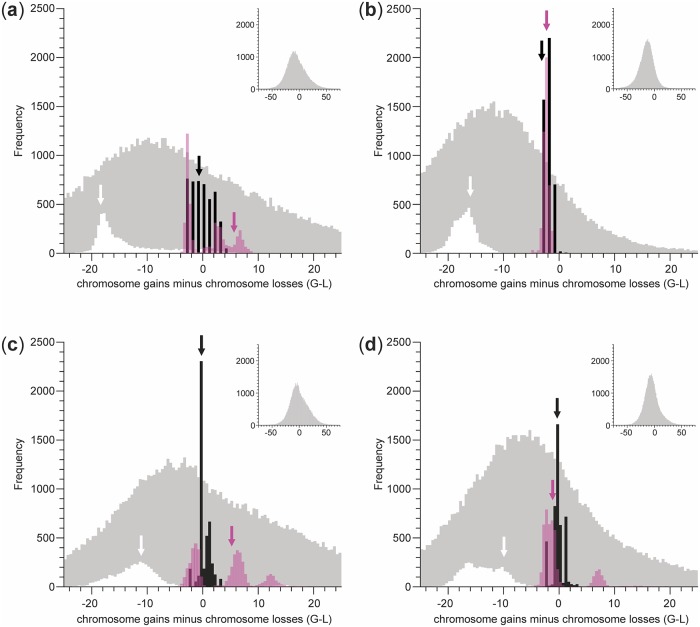
Distributions of the number of chromosome gains minus the number of chromosome losses (G-L) in *Melampodium*. G-L distributions reconstructed on phylogenetic trees obtained from analyses of (A) nuclear sequence data using BEAST (ITS-B), (B) nuclear sequence data using MrBayes (ITS-MB), (C) plastid sequence data using BEAST (matK-B) and (D) plastid sequence data using MrBayes (matK-MB). Methods of chromosome number reconstruction are indicated by colors: black—ordered maximum parsimony (MP); purple—maximum likelihood using ChromEvol (ML-CE; results are shown for the Constant-Rate No Duplication (CRND) model); white—maximum likelihood using BayesTraits (ML-BT; results are shown for the *two-rate* model); grey—Bayesian Inference (BI; results are shown for the *two-rate* model). Arrows indicate positions of the Maximum Clade Credibility (MCC) trees. Inserts show the full G-L distributions from the BI analysis, which are truncated in the main figure to aid legibility.

Results are summarized in Table 6 and in Fig 2. As for the ML-CE analyses in the majority of cases the model with no duplication incorporating dysploid change at a constant rate (CRND) was the best fit (Table 1), all comparisons were based on the CRND model (for ML-CE) and the *two-rate* model (for ML-BT and BI). Variances of G-L distributions were smallest in the MP analysis, largest in the BI analysis, and intermediate in the ML-CE and ML-BT analyses. Within the same analysis method, these variances were larger for the BEAST data set than for the MrBayes data set (except for matK-B and matK-MB in the MP analysis, where they were essentially identical), but the narrower G-L distributions from the MrBayes analyses were always (nearly) completely nested within the broader G-L distributions from the BEAST analyses. Compared to MP and ML-CE analyses, the means of the G-L distributions from the ML-BT and particularly the BI analyses were strongly shifted towards smaller values. A potential cause for this apparent bias towards loss is that a character state must be observed in the tips to be considered for the ancestral states by BayesTraits, hence neither taking intermediate, but unobserved chromosome numbers (*n* = 13 in case of *Melampodium*) nor chromosome numbers outside the range of observed numbers into account. To investigate this, additional data sets were constructed, where *M. repens*, the single species with *n* = 14, was pruned from the trees; these data sets were then analyzed with the CRND model, where for ChromEvol additionally minimum and maximum chromosome number were set to 9 and 12, respectively, to enforce identical dimensions of the transition matrices for both programs. G-L distributions from these reduced data sets were shifted towards more losses only in the analyses using ChromEvol, but variances were reduced irrespective of program used (S1 Fig).

**Table 6:** Characteristics of the distribution of chromosome gains minus chromosome losses (G-L).

Dataset	Method	Mean / Median (Range)	Mode	MCC Tree
ITS-B	MP	-0.341 / 0.000 (-4.000–4.000)	-3.000	-1.000
	ML-CE	0.802 / -0.414 (-5.012–15.900)	-2.615	6.365
	ML-BT	-13.080 / -17.960 (-29.050–25.700)	-17.949	-19.394
	BI	-6.222 / -7.956 (-188.589–151.621)	-8.253	n.a.
ITS-MB	MP	-2.179 / -2.000 (-3.000–2.000)	-2.000	-3.000
	ML-CE	-2.348 / -2.314 (-5.022–0.826)	-2.400	-2.474
	ML-BT	-17.021 / -17.131 (-48.6761–-0.799)	-16.476	-16.116
	BI	-15.262 / -14.203 (-131.142–70.216)	-11.441	n.a.
matK-B	MP	0.1300 / -0.330 (-2.300–5.670)	-0.305	-0.330
	ML-CE	3.912 / 5.405 (-4.602–18.620)	-1.278	5.924
	ML-BT	-9.952 / -11.697 (-27.108–52.297)	-10.986	-10.506
	BI	-0.737 / -2.622 (-163.757–130.269)	-7.996	n.a.
matK-MB	MP	-0.327 / -0.330 (-2.330–3.990)	-0.239	-1.330
	ML-CE	-0.229 / -1.500 (-5.233–18.300)	-2.262	-0.743
	ML-BT	-12.690 / -13.720 (-24.560–31.230)	-16.276	-13.940
	BI	-4.957 / -5.899 (-106.285–91.412)	-5.303	n.a.

Each data set (abbreviations as in Table 1) has been analyzed using each of four methods (MP—ordered Maximum Parsimony; ML-CE—Maximum Likelihood using ChromEvol; ML-BT—Maximum Likelihood using BayesTraits; BI-RJ—Bayesian Inference using Reversible Jump). For the distributions of the test statistic G-L (difference between chromosome gains and chromosome losses) mean, median, range and mode (calculated using the Chernoff mode estimator with bandwidth of 0.5 as implemented in the R package modeest) are given as well as the G-L values for the maximum clade credibility (MCC) trees.

The G-L distributions from ML-CE analyses of ITS-B, matK-MB and maK-B were multi-modal (Fig 2). Although multi-modality was also observed for ML-BT, it was much weaker and a single peak—the one visible in Fig 2—dominated; multi-modality became, however, more pronounced in the truncated data set (S1 Fig). The different modes were found to be highly correlated with the inferred root state (S2 Fig). Although multi-modality was not restricted to the ultrametric trees (ITS-B, matK-B), the effect was more pronounced in comparison to analyses using the phylograms (ITS-MB, matK-MB; Fig 2). To test whether ultrametricization per se contributes to multi-modality, the original MrBayes phylograms were ultrametricized using PATHd8 [58] and then analyzed with the CRND model used for the original data. Indeed, ultrametricization resulted in either the introduction of multi-modality (ITS-MB) or an accentuation of already existing multi-modality (matK-MB; S3 Fig).

BEAST trees differed significantly from MrBayes trees in their imbalance (Table 7), measured using Colless’ Imbalance Index [59] calculated using Mesquite 2.75. Specifically, BEAST trees were more balanced (had smaller index values) than MrBayes trees (one-tailed Wilcoxon rank-sum test, conducted with the function Wilcox.test in R [60]: W = 5533400, *p* < 0.001, for ITS-B versus ITS-MB; W = 15720000, *p* < 0.001, for matK-B versus matK-MB). Likewise, BEAST trees differed significantly from MrBayes trees in their stemminess (Table 7), measured using the non-cumulative stemminess index of Rohlf et al. [61]. Specifically, the ultrametric BEAST trees were less stemmy than the corresponding MrBayes trees (one-tailed Wilcoxon rank-sum test W = 9858475, *p* = 0.0153 for ITS-B versus ITS-MB; W = 4034709, *p* < 0.001 for matK-B versus matK-MB). Thus, a possible underlying cause for the effect of ultrametricization might be a decrease in stemminess. To test this, the stemminess of the non-ultrametricized trees (ITS-MB, matK-MB) was compared to that of their ultrameticized counterparts using one-tailed Wilcoxon signed-rank tests (Table 7). However, while stemminess decreased for the matK data set (V = 3272320, *p* < 0.001), it increased for the ITS data set (V = 7075097, *p* < 0.001).

**Table 7:** Colless Imbalance and Stemminess Indices (given as mean / median and, in parentheses, range) of the phylogenetic trees used in the analyses.

Dataset	Colless Imbalance	Stemminess	
		Original	Ultrametricised
ITS-B	0.188 / 0.191 (0.084–0.350)	1.487 / 0.604 (0.104–288.930)	
ITS-MB	0.230 / 0.243 (0.115–0.316)	1.302 / 0.589 (0.264–341.094)	1.086 / 0.672 (0.241–262.604)
matK-B	0.162 / 0.153 (0.106–0.311)	1.453 / 0.668 (0.148–689.416)	
matK-MB	0.188 / 0.189 (0.117–0.292)	4.687 / 1.279 (0.346–2293.74)	2.567 / 0.809 (0.239–799.423)

For the trees of each data set (abbreviations as in Table 1) Colless Imbalance and Stemminess have been calculated (see text for details), the latter both for trees with the original branch lengths (column “Original”) and (in case of trees obtained from MrBayes) for trees, whose branch lengths have been modified so that trees become ultrametric using PATHd8 (column “Ultrametricized”).

### MCC trees

For data sets with low model uncertainty (ITS-B, matK-MB, matK-B) in the ML-CE analyses, the best-supported model for the MCC tree was the one best-supported over all, but in case of the data set ITS-MB, where model uncertainty was high, the best-supported model for the MCC tree was only the second-best supported over all. For the ML-BT analyses, where model uncertainty was much lower, the best-supported model for the MCC trees was the one best-supported over all.

For the majority of nodes, node-wise reconstruction precision (*RP*_*n*_) on the MCC tree was a good representation of *RP*_*n*_ integrated over all trees (shown on the consensus tree in Fig 1), deviations usually being less than 10% (S7 Table). Most exceptions were overestimating reconstruction precision (up to nearly 45%), a few were underestimating it (twice in BT-ML up to 30%, twice in MP up to 60%). Corresponding to the good fit in node-wise reconstruction uncertainty, tree-wide reconstruction uncertainty, *RP*_*t*_, estimated on the MCC tree deviated from *RP*_*t*_ estimated on the majority rule consensus tree by less than 5% (Table 5).

In MP reconstructions, the MCC tree was close to or at the mode of the G-L distribution (Fig 2, Table 6). This is expected, because clades found in the MCC tree will tend to be those with high posterior probabilities, i.e., they are frequently present also in other posterior trees. Whereas under ordered parsimony the location of the MCC tree in the G-L distribution did not change much across data sets, it was much more variable in the ML-CE and BT-ML analyses (Fig 2). Multi-modality of G-L distributions negatively affected the suitability of the MCC tree as single representative of the posterior distribution. This was particularly pronounced in ITS-B, where the MCC was located off both the mode and the mean of the G-L distribution (Fig 2, Table 6). Additionally, multi-modality could not be appropriately described by commonly used measures, such as the mean or the confidence interval, especially if distributions were (nearly) discontinuous as was the case for matK-MB (Fig 2).

### Chromosome number evolution in *Melampodium*

The most frequently inferred ancestral chromosome number was *n* = 11, either unambiguously (with probabilities above 0.8; ITS-MB and matK-MB) or ambiguously (with probabilities mostly below 0.8 down to less than 0.5) together with *n* = 10 (ITS-B and matK-B; Fig 1). Irrespective of these uncertainties, chromosome numbers in *Melampodium* represented a bidirectional dysploid series with neither ascending nor ascending dysploidy dominating (most G-L distributions contained zero: Fig 2).

## Discussion

### Chromosome number reconstruction under phylogenetic uncertainty

In evolutionary studies employing ancestral character state reconstruction it is common practice to use a summary of the posterior distribution of trees from a Bayesian analysis, usually the Majority-Rule Consensus (MRC) tree, or a single representative of these posterior trees, such as the Maximum a posteriori (MAP) tree or the Maximum Clade Credibility (MCC) tree [27, 62]. Of those, the MCC tree is most commonly used, because it contains information on branch lengths. As shown here, ignoring phylogenetic uncertainty and using a single tree, even the one with highest clade credibility, can be misleading. This is particularly pronounced for the derived statistics of the number of chromosome gains minus chromosome losses (G-L). Here, the position of the MCC tree in the G-L distributions was unpredictable, especially in multimodal distributions, and inconsistent across tree datasets (Fig 2). The discrepancy between inferences of ancestral chromosome numbers on the MCC tree from those made by integrating over a set of trees were less severe, especially in reconstructions made with ChromEvol (Table 5).

A second consequence of using a single representative tree is that model uncertainty will be underestimated, if the best-fit model differs among trees, e.g., those in the set of posterior trees from a Bayesian analysis (Tables 1 and 3). This and model selection uncertainty (i.e., the confidence in the selected model compared to the other candidate models; Tables 2 and 4) can be readily accounted for by model averaging [54] using, for instance, Akaike or Schwarz weights (calculated from AIC and BIC, respectively) in a maximum likelihood framework [63] or reversible-jump MCMC [57, 64] in a Bayesian framework (note that model averaging as used here does not address model heterogeneity, i.e., different best models for different nodes of the same tree: [65]). The current implementation of reversible-jump MCMC in BayesTraits does not allow reduction of model space a priori (for instance, to the three models used in the maximum likelihood analysis of BayesTraits). Consequently, real data sets may be too small to contain sufficient signal to allow decisive discrimination among models. This is likely the case for the present *Melampodium* data set, where nearly none of the rates of dysploid change could be rejected with confidence (S5 Table). In summary, reliance on single trees, such as MCC trees, is strongly discouraged not only for chromosome number reconstruction (as shown here), but for ancestral character state reconstruction in general as has been repeatedly suggested before [30, 66–68].

An important source of parameter variability is the branch length model, i.e., whether phylograms or ultrametric trees are used [23, 69]. In *Melampodium*, this was evident from broader ranges of G-L and a tendency towards multi-modality of G-L distributions (Fig 2, Table 6), resulting in higher uncertainty with respect to ancestral chromosome numbers (Fig 1, Table 5) when using ultrametric trees. These differences are due to ultrametricization per se (S3 Fig), but not due to higher phylogenetic uncertainty in the posterior set obtained from BEAST (number of unique topologies 4476 and 4443 in ITS-B and ITS-MB, respectively, but 1929 and 2372 in matK-B and matK-MB, respectively). Cusimano and Renner [23] reported that total tree length, tree imbalance and particularly tree stemminess contributed to such differences. Trees in our analyses were scaled to equal length prior to analyses, hence total tree length cannot account for the observed pattern; this may also explain why we did not find any consistent relationship with changes in stemminess (Table 7; Cusimano and Renner [23] did not rescale trees to equal total tree length). Tree imbalance alone cannot explain the observed differences either, as it does not differ between the MrBayes phylograms and their ultrametricized counterparts. Although additional, yet untested, tree features that change between a phylogram and an ultrametric tree might be responsible for these discrepancies, it is more likely that these discrepancies are the result of a combination of tree features (e.g., stemminess) and data features (e.g., frequency and distribution of character states on the tree). Testing this hypothesis will, however, require extensive simulation studies.

There is no ready answer whether reconstructions of multistate characters on phylograms should be preferred over those on ultrametric trees or vice versa [23]. If it cannot be clearly established whether a given character (here chromosome number) evolves proportional to time (i.e., in a clock-like manner) or proportional to genetic distance (i.e., correlating with molecular evolution), then both types of trees may be used. If results differ, external evidence such as fossil data or, in the context of chromosome number evolution, cytological evidence for chromosome number altering chromosome rearrangements [70, 71] can help to decide among competing scenarios (that may also result from sources other than different branch length models, e.g., likelihood versus parsimony reconstructions).

Ordered maximum parsimony analyses consistently show the least amount of variation within and among data sets compared to model-based approaches (Figs 1 and 2, Tables 5 and 6). This is expected given that maximum parsimony reconstructions will only be affected by changes in tree topology and not by differences in branch lengths [21]. Although maximum parsimony reconstructions tend to underestimate the amount of character state change [21], such a bias may be less severe for chromosome numbers, if anagenetic changes in chromosome number are rare and changes in chromosome number are frequently connected to speciation (likely via similar mechanisms as suggested for chromosomal speciation via inversions: [72–74]). Thus, just as Fitch parsimony has been suggested to be an appropriate model in biogeography when dispersal rates are low [75], ordered maximum parsimony continues to be a valid model when studying chromosome number changes.

Maximum likelihood reconstructions of chromosome numbers differ little between BayesTraits and ChromEvol (BayesTraits reconstructions tend to be associated with higher uncertainty, if averaged over a set of trees: Fig 1, Table 5), but this is not the case for the inferred gains and losses and their difference (Fig 2). As we compared the *two-rate* model of BayesTraits with a model of ChromEvol that did include neither duplications nor demi-duplications, this difference in model parameterization cannot explain the discordant results. Instead, they may be due to the different implementations of continuous-time Markov models in the two programs. In contrast to BayesTraits, ChromEvol can take unobserved character states both within and outside the range of observed chromosome numbers into account [29]. Although results from BayesTraits become more similar to those of ChromEvol once character space is contiguous (i.e., after exclusion of the single taxon with *n* = 14; S1 Fig) and upper and lower bounds (i.e., maximum and minimum observed number) are the same for both methods, the results concerning the difference of gains and losses remain incompatible (Fig 2), indicating that additional factors are responsible for the observed discrepancies. It remains to be tested whether the usability of BayesTraits (including its Bayesian implementation) in the context of studying chromosome number evolution may be limited. Although not tested here, the model limitations described for BayesTraits also apply to stochastic character mapping [76, 77] as currently implemented (e.g., in Simmap: [78]); this method has, however, only been rarely used for chromosome number reconstruction [79].

### Chromosome number evolution in *Melampodium*

Both *x* = 10 and *x* = 11 have been proposed as ancestral chromosome base number for *Melampodium*. Support for *x* = 10 came from higher morphological diversity, higher species number, and the presence of a conspicuously demarcated sterile ovary in the disc florets, a presumably primitive character, in *Melampodium* with *x* = 10 [34, 38]. On the other hand, the presence of *x* = 11 in the closest relatives of *Melampodium*, *Acanthospermum* and *Lecocarpus*, suggested *x* = 11 as ancestral chromosome base number [34]. This latter hypothesis is supported by the present analyses, although not unambiguously when time-calibrated trees are used (Fig 1).

Chromosome number evolution in *Melampodium* follows a pattern of bidirectional dysploidy (Fig 1) with no prevailing direction (as evident from G-L distributions containing zero and the *one-rate* model being the best supported in BayesTraits: Fig 2, Table 3). In plants, descending dysploid series have been suggested to be more common than ascending ones [80, 81]. A prevalence of descending dysploidy may be expected, because genome diploidization after polyploidization is often associated with a reduction in chromosome number [82]. As shown here for *Melampodium* and known for other Asteraceae and beyond [6, 14, 83], a view of a unidirectional progressive dysploid series likely is too simplistic [1].

Change of chromosome base number in *Melampodium* may have contributed to lineage divergence, for instance via accelerated genic diversification following chromosomal rearrangements [74, 84]. Judging from species numbers, only the change to *x* = 10 might have had an effect on lineage diversification: sect. *Melampodium* with *x* = 10 contains more than half of the *Melampodium* species, while lineages possessing *x* = 9 (sect. *Zarabellia*), 12 (sect. *Serratura*), or 14 (sect. *Bibractiaria*) each comprise only one or a few diploid species [34]. It remains to be tested whether the dysploid change per se or correlated factors could have affected lineage diversification in *Melampodium*.

## Supporting Information

S1 Fig Distributions of the number of chromosome gains minus the number of chromosome losses (G-L) with and without *Melampodium repens*. G-L distributions reconstructed using maximum likelihood in ChromEvol (ML-CE, grey) and in BayesTraits (ML-BT, shades of red) on phylogenetic trees obtained from analyses of (A) nuclear sequence data using BEAST (ITS-B), (B) nuclear sequence data using MrBayes (ITS-MB), (C) plastid sequence data using BEAST (matK-B) and (D) plastid sequence data using MrBayes (matK-MB) before (dark grey and orange) and after (light grey and red) pruning *Melampodium repens*, the sole species with *n* = 14, and restricting minimum and maximum chromosome number to 9 and 12, respectively.
(PDF)

S2 Fig Relationship between root state and the number of chromosome gains minus the number of chromosome losses (G-L. Relationship between root state and G-L reconstructed using maximum likelihood in ChromEvol (ML-CE) on phylogenetic trees obtained from analyses of (A) nuclear sequence data using BEAST (ITS-B), (B) nuclear sequence data using MrBayes (ITS-MB), (C) plastid sequence data using BEAST (matK-B) and (D) plastid sequence data using MrBayes (matK-MB).
(PDF)

S3 Fig Distributions of the number of chromosome gains minus the number of chromosome losses (G-L) before and after ultrametricization. G-L distributions reconstructed using maximum likelihood in ChromEvol (ML-CE) on phylogenetic trees obtained from analyses of (A) nuclear sequence data using MrBayes (ITS-MB) and (B) plastid sequence data using MrBayes (matK-MB) before (black) and after (grey) ultrametricization using PATHd8.
(PDF)

S1 Table Species names, chromosome numbers, localities, voucher numbers and GenBank accession numbers of the analyzed taxa. Chromosome numbers of polyploid cytotypes (not used in this study) are given in parentheses. Collection details are given in the following format: Locality and year; Collector (Herbarium: Collection Number); herbaria are WU and MEXU, unless otherwise indicated. The outgroup taxa were used for rooting of phylogenetic trees obtained with MrBayes, but were removed from chromosome number reconstruction.
(XLSX)

S2 Table Minimum and maximum ΔAICs of the Linear Rate models against the best model. Each data set (ITS-B—nuclear sequence data analyzed using BEAST; ITS-MB—nuclear sequence data set analyzed using MrBayes; matK-B—plastid sequence data analyzed using BEAST; matK-MB—plastid sequence data analyzed using MrBayes) has been analyzed under each of eight models implemented in ChromEvol 2. The best supported model has been compared against the models including a dependency of dysploid change on chromosome number (LRND—Linear Rate—No Duplication; LRD—Linear Rate—Duplication only; LRDD—Linear Rate—identical Demi-duplication and Duplication; LRDE—Linear Rate—Demi-duplication Estimated; see main text for details).
(XLSX)

S3 Table Minimum and maximum ΔAICs of the *multi-rate* model against the best model. Each data set (abbreviations as in S2 Table) has been analyzed under each of three models implemented in BayesTraits 2. The best supported model has been compared against the *multi-rate* model (see main text for details).
(XLSX)

S4 Table Model comparison of *one-rate*, *two-rate* and *multi-rate* model used in a Bayesian analysis (BI). Each data set (abbreviations as in S2 Table) has been analyzed in a Bayesian framework under each of three models implemented in BayesTraits 2; models have been compared using BayesFactors.
(XLSX)

S5 Table Number of rate classes, number of rates being zero and distribution of rates in rate class zero from the Bayesian Reversible-Jump (BT-RJ) analyses. Each data set (abbreviations as in S2 Table) has been analyzed in a Bayesian framework using reversible jump implemented in BayesTraits 2. Number of rate classes, given as mean (range), and the number of rates being 0, given as mean (range), provide information on model uncertainty. The proportion of the focal rate (given as ancestral chromosome number → derived chromosome number) provides information on the importance of the respective rates; values in bold are proportions smaller than 0.05.
(XLSX)

S6 Table Correlation between integrated node-wise reconstruction precision (*RP*_*n*_) and individual reconstruction precision. Node-wise reconstruction precision, *RP*_*n*_, has been calculated for those nodes (C0 to C23) present in at least 95% of posterior trees per analysis and data set (abbreviations of data sets as in S2 Table) by integrating over the input trees (integrated *RP*_*n*_). For each of these nodes, the proportion of trees, where node-wise reconstruction precision is at or above a certain threshold (0.9 and 0.95, respectively), has been recorded (individual *RP*_*n*_). If low integrated reconstruction precision is due to to ambiguous reconstructions in the input trees (resulting in small *RP*_*n*_s in each tree), a close correlation between integrated and individual *RP*_*n*_ is expected. If low integrated reconstruction precision is due to to unambiguous but contradicting reconstructions in the input trees (resulting in *RP*_*n*_s close to 1 in each tree), no correlation between integrated and individual *RP*_*n*_ is expected. These expectations have been tested using Pearson’s correlation coefficients.
(XLSX)

S7 Table Comparison of node-wise reconstruction precision (*RP*_*n*_) of separate trees, integrated over all trees and shown on the consensus tree, and of the Maximum Clade Credibility (MCC) tree. Node-wise reconstruction precision, *RP*_*n*_, has been calculated for those nodes (C0 to C23) present in at least 95% of posterior trees per analysis and data set (abbreviations of data sets as in S2 Table). The fit of the nodewise reconstruction precision integrated over all tree (columns “consensus”) and the nodewise reconstruction precision of the maximum clade credibility tree (column “MCC”) has been assessed by their ratio (column “ratio MCC / consensus”). Overestimation and underestimation by 10% or more of node-wise reconstruction precision on the MCC tree are indicated in red and blue, respectively.
(XLSX)

## References

[1] Weiss-Schneeweiss H, Schneeweiss GM. Karyotype diversity and evolutionary trends in angiosperms In: Leitch IJ, Greilhuber J, Doležel J, Wendel J, editors. Plant Genome Diversity Vol. 2. Wien: Springer; 2013 p. 209–230.

[2] Escudero M, Martín-Bravo S, Mayrose I, Ferná¡ndez-Mazuecos M, Fiz-Palacios O, Hipp AL, et al Karyotypic changes through dysploidy persist longer over evolutionary time than polyploid changes. PLoS ONE. 2014;9(1):e85266 10.1371/journal.pone.0085266 24416374 PMC3887030

[3] de Storme N, Mason A. Plant speciation through chromosome instability and ploidy change: Cellular mechanisms, molecular factors and evolutionary relevance. Curr Plant Biol. 2014;1:10–33. 10.1016/j.cpb.2014.09.002

[4] Stebbins GL. Chromosomal evolution in higher plants. London: Edward Arnold; 1971.

[5] Levin DA. The role of chromosomal change in plant evolution. New York: Oxford University Press; 2002.

[6] Samuel R, Stuessy TF, Tremetsberger K, Baeza CM, Siljak-Yakovlev S. Phylogenetic relationships among species of *Hypochaeris* (Asteraceae, Cichorieae) based on ITS, plastid trnL intron, trnL-F spacer, and matK sequences. Am J Bot. 2003;90(3):496–507. 10.3732/ajb.90.3.496 21659142

[7] Hansen AK, Gilbert LE, Simpson BB, Downie SR, Cervi AC, Jansen RK. Phylogenetic relationships and chromosome number evolution in *Passiflora*. Syst Bot. 2006;31(1):138–150. 10.1600/036364406775971769

[8] Schneeweiss GM, Pachschwöll C, Tribsch A, Schönswetter P, Barfuss MH, Esfeld K, et al Molecular phylogenetic analyses identify Alpine differentiation and dysploid chromosome number changes as major forces for the evolution of the European endemic *Phyteuma* (Campanulaceae). Mol Phylogenet Evol. 2013;69(3):634–652. 10.1016/j.ympev.2013.07.015 23891952

[9] Jiao Y, Wickett NJ, Ayyampalayam S, Chanderbali AS, Landherr L, Ralph PE, et al Ancestral polyploidy in seed plants and angiosperms. Nature. 2011;473 10.1038/nature09916 21478875

[10] Kagale S, Robinson SJ, Nixon J, Xiao R, Huebert T, Condie J, et al Polyploid evolution of the Brassicaceae during the Cenozoic Era. The Plant Cell. 2014;26:2777–2791. 10.1105/tpc.114.126391 25035408 PMC4145113

[11] Weiss-Schneeweiss H, Emadzade K, Jang TS, Schneeweiss GM. Evolutionary consequences, constraints and potential of polyploidy in plants. Cytogenet Genome Res. 2013;140(2–4):137–150. 10.1159/000351727 23796571 PMC3859924

[12] Leitch AR, Leitch IJ. Genomic plasticity and the diversity of polyploid plants. Science. 2008;320(5875):481–483. 10.1126/science.1153585 18436776

[13] Hipp AL. Nonuniform processes of chromosome evolution in sedges (*Carex*: Cyperaceae). Evolution. 2007;61(9):2175–2194. 10.1111/j.1558-5646.2007.00183.x 17767589

[14] Enke N, Gemeinholzer B. Babcock revisited: new insights into generic delimitation and character evolution in *Crepis* L. (Compositae: Cichorieae) from ITS and matK sequence data. Taxon. 2008;57(3):756–768.

[15] Jang TS, Emadzade K, Parker J, Temsch EM, Leitch AR, Speta F, et al Chromosomal diversification and karyotype evolution of diploids in the cytologically diverse genus *Prospero* (Hyacinthaceae). BMC Evol Biol. 2013;13(1):136 10.1186/1471-2148-13-136 23819574 PMC3728210

[16] Rice A, Glick L, Abadi S, Einhorn M, Kopelman NM, Salman-Minkov A, et al The Chromosome Counts Database (CCDB)–a community resource of plant chromosome numbers. New Phytol. 2015;206(1):19–26. 10.1111/nph.13191 25423910

[17] Stuessy TF, Crawford DJ, Soltis DE, Soltis PS. Plant systematics: The origin, interpretation, and ordering of plant biodiversity. Königstein: Koeltz Scientific Books; 2014.

[18] Beardsley PM, Schoenig SE, Whittall JB, Olmstead RG. Patterns of evolution in western North American *Mimulus* (Phrymaceae). Am J Bot. 2004;91(3):474–489. 10.3732/ajb.91.3.474 21653403

[19] Hennequin S, Ebihara A, Dubuisson JY, Schneider H. Chromosome number evolution in *Hymenophyllum* (Hymenophyllaceae), with special reference to the subgenus *Hymenophyllum*. Mol Phylogenet Evol. 2010;55(1):47–59. 10.1016/j.ympev.2010.01.001 20060917

[20] Schubert I, Lysak MA. Interpretation of karyotype evolution should consider chromosome structural constraints. Trends Genet. 2011;27(6):207–216. 10.1016/j.tig.2011.03.004 21592609

[21] Cunningham CW, Omland KE, Oakley TH. Reconstructing ancestral character states: a critical reappraisal. Trends Ecol Evol. 1998;13(9):361–366. 10.1016/S0169-5347(98)01382-2 21238344

[22] Cunningham CW. Some limitations of ancestral character-state reconstruction when testing evolutionary hypotheses. Syst Biol. 1999;48(3):665–674. 10.1080/106351599260238

[23] Cusimano N, Renner SS. Ultrametric trees or phylograms for ancestral state reconstruction: does it matter? Taxon. 2014;63(4):721–726.

[24] Pagel M. The maximum likelihood approach to reconstructing ancestral character states of discrete characters on phylogenies. Syst Biol. 1999;48:612–622. 10.1080/106351599260184

[25] Kadane JB, Lazar NA. Methods and criteria for model selection. J Am Stat Assoc. 2004;99:279–290. 10.1198/016214504000000269

[26] Lewis PO. A likelihood approach to estimating phylogeny from discrete morphological character data. Syst Biol. 2001;50(6):913–925. 10.1080/106351501753462876 12116640

[27] Pagel M, Meade A, Barker D. Bayesian estimation of ancestral character states on phylogenies. Syst Biol. 2004;53:673–684. 10.1080/10635150490522232 15545248

[28] Mayrose I, Barker MS, Otto SP. Probabilistic models of chromosome number evolution and the inference of polyploidy. Syst Biol. 2010;59(2):132–144. 10.1093/sysbio/syp083 20525626

[29] Glick L, Mayrose I. ChromEvol: assessing the pattern of chromosome number evolution and the inference of polyploidy along a phylogeny. Mol Biol Evol. 2014;31(7):1914–1922. 10.1093/molbev/msu122 24710517

[30] Duchêne S, Lanfear R. Phylogenetic uncertainty can bias the number of evolutionary transitions estimated from ancestral state reconstruction methods. J Exp Zool Part B: Mol Dev Evol. 2015;324(6):517–524. 10.1002/jez.b.22638 26173578

[31] Pellicer J, Kelly LJ, Leitch IJ, Zomlefer WB, Fay MF. A universe of dwarfs and giants: genome size and chromosome evolution in the monocot family Melanthiaceae. New Phytol. 2014;201(4):1484–1497. 10.1111/nph.12617 24299166

[32] Chacón J, Cusimano N, Renner SS. The evolution of Colchicaceae, with a focus on chromosome numbers. Syst Bot. 2014;39(2):415–427. 10.1600/036364414X680852

[33] Sousa A, Renner SS. Interstitial telomere-type repeats in the monocot family Araceae. Bot J Linn Soc. 2015;177:15–26. 10.1111/boj.12231

[34] Stuessy TF, Blöch C, Villaseñor JL, Rebernig CA, Weiss-Schneeweiss H. Phylogenetic analyses of DNA sequences with chromosomal and morphological data confirm and refine sectional and series classification within *Melampodium* (Asteraceae, Millerieae). Taxon. 2011;60(2):436–449.

[35] Weiss-Schneeweiss H, Stuessy TF, Villaseñor JL. Chromosome numbers, karyotypes, and evolution in *Melampodium* (Asteraceae). Int J Plant Sci. 2009;170(9):1168–1182. 10.1086/605876

[36] Weiss-Schneeweiss H, Blöch C, Turner B, Villaseñor JL, Stuessy TF, Schneeweiss GM. The promiscuous and the chaste: frequent allopolyploid speciation and its genomic consequences in American daisies (*Melampodium* sect. *Melampodium*; Asteraceae). Evolution. 2012;66(1):211–228. 10.1111/j.1558-5646.2011.01424.x 22220876

[37] Blöch C, Weiss-Schneeweiss H, Schneeweiss GM, Barfuss MH, Rebernig CA, Villaseñor JL, et al Molecular phylogenetic analyses of nuclear and plastid DNA sequences support dysploid and polyploid chromosome number changes and reticulate evolution in the diversification of *Melampodium* (Millerieae, Asteraceae). Mol Phylogenet Evol. 2009;53(1):220–233. 10.1016/j.ympev.2009.02.021 19272456 PMC4268500

[38] Stuessy TF. Chromosome numbers and phylogeny in *Melampodium* (Compositae). Am J Bot. 1971;58(8):732–736. 10.2307/2441471

[39] Keil DJ, Luckow MA, Pinkava DJ. Chromosome studies in Asteraceae from the United States, Mexico, the West Indies, and South America. Am J Bot. 1988;75:652–668. 10.2307/2444199 30139084

[40] Tamura K, Dudley J, Nei M, Kumar S. MEGA4: molecular evolutionary genetics analysis (MEGA) software version 4.0. Mol Biol Evol. 2007;24(8):1596–1599. 10.1093/molbev/msm092 17488738

[41] Posada D, Crandall KA. Modeltest: testing the model of DNA substitution. Bioinformatics. 1998;14:817–818. 10.1093/bioinformatics/14.9.817 9918953

[42] Yang Z. Molecular evolution a statistical approach. Oxford: Oxford Univ. Press; 2014.

[43] Ronquist F, Huelsenbeck JP. MrBayes 3: Bayesian phylogenetic inference under mixed models. Bioinformatics. 2003;19(12):1572–1574. 10.1093/bioinformatics/btg180 12912839

[44] Brown JM, Hedtke SM, Lemmon AR, Lemmon EM. When trees grow too long: Investigating the causes of highly inaccurate Bayesian branch-length estimates. Syst Biol. 2010;59(2):145–161. 10.1093/sysbio/syp081 20525627

[45] Canne JM. Cytological and morphological observations in *Galinsoga* and related genera (Asteraceae). Rhodora. 1977;85(843):355–366.

[46] Turner BL, Triplett K. Revisionary study of the genus *Milleria* (Asteraceae, Heliantheae). Phytologia. 1996;81(5):348–360.

[47] Drummond AJ, Rambaut A. BEAST: Bayesian evolutionary analysis by sampling trees. BMC Evol Biol. 2007;7(1):214 10.1186/1471-2148-7-214 17996036 PMC2247476

[48] Kuzoff RK, Sweere JA, Soltis DE, Soltis PS, Zimmer EA. The phylogenetic potential of entire 26S rDNA sequences in plants. Mol Biol Evol. 1998;15(3):251–263. 10.1093/oxfordjournals.molbev.a025922 9501492

[49] Bremer K, Gustafsson MHG. East Gondwana ancestry of the sunflower alliance of families. Proc Natl Acad Sci USA. 1997;94(17):9188–9190. 10.1073/pnas.94.17.9188 9256457 PMC23106

[50] Kay KM, Whittall JB, Hodges SA. A survey of nuclear ribosomal internal transcribed spacer substitution rates across angiosperms: an approximate molecular clock with life history effects. BMC Evol Biol. 2006;6(1):1–9. 10.1186/1471-2148-6-36 16638138 PMC1484492

[51] Yamane K, Yasui Y, Ohnishi O. Intraspecific cpDNA variations of diploid and tetraploid perennial buckwheat, *Fagopyrum cymosum* (Polygonaceae). Am J Bot. 2003;90(3):339–346. 10.3732/ajb.90.3.339 21659125

[52] Maddison W, Maddison D. Mesquite: a modular system for evolutionary analysis. Version 2.75.; 2011. http://mesquiteproject.org.

[53] Sukumaran J, Holder MT. DendroPy: a Python library for phylogenetic computing. Bioinformatics. 2010;26(12):1569–1571. 10.1093/bioinformatics/btq228 20421198

[54] Posada D, Buckley TR. Model selection and model averaging in phylogenetics: advantages of Akaike Information Criterion and Bayesian approaches over likelihood ratio tests. Syst Biol. 2004;53(5):793–808. 10.1080/10635150490522304 15545256

[55] Kass RE, Raftery AE. Bayes Factors. J Am Stat Assoc. 1995;90:773–795. 10.1080/01621459.1995.10476572

[56] Baele G, Lemey P, Bedford T, Rambaut A, Suchard MA, Alekseyenko AV. Improving the accuracy of demographic and molecular clock model comparison while accommodating phylogenetic uncertainty. Mol Biol Evol. 2012;29(9):2157–2167. 10.1093/molbev/mss084 22403239 PMC3424409

[57] Pagel M, Meade A. Bayesian analysis of correlated evolution of discrete characters by reversible-jump Markov chain Monte Carlo. Am Nat. 2006;167(6):808–825. 10.1086/503444 16685633

[58] Britton T, Anderson CL, Jacquet D, Lundqvist S, Bremer K. Estimating divergence times in large phylogenetic trees. Syst Biol. 2007;56(5):741–752. 10.1080/10635150701613783 17886144

[59] Colless DH. Review of: Phylogenetics: the theory and practice of phylogenetic systematics. Syst Biol. 1982;31:100–104.

[60] R Core Team. R: A language and environment for statistical computing; 2015. https://www.R-project.org.

[61] Rohlf FJ, Chang WS, Sokal RR, Kim J. Accuracy of estimated phylogenies: Effects of tree topology and evolutionary model. Evolution. 1990;44(6):1671–1684. 10.2307/2409346 28564306

[62] Heled J, Bouckaert RR. Looking for trees in the forest: summary tree from posterior samples. BMC Evol Biol. 2013;13:221 10.1186/1471-2148-13-221 24093883 PMC3853548

[63] Burnham KP, Anderson DR. Multimodal inference: understanding AIC and BIC in model selection. Sociol Meth Res. 2004;33(2):261–304. 10.1177/0049124104268644

[64] Pagel M, Meade A. Modelling heterotachy in phylogenetic inference by reversible-jump Markov chain Monte Carlo. Philos Trans R Soc Lond B Biol Sci. 2008;363(1512):3955–3964. 10.1098/rstb.2008.0178 18852097 PMC2607421

[65] Royer-Carenzi M, Pontarotti P, Didier G. Choosing the best ancestral character state reconstruction method. Math Biosci. 2013;242(1):95–109. 10.1016/j.mbs.2012.12.003 23276531

[66] Huelsenbeck JP, Bollback JP. Empirical and hierarchical Bayesian estimation of ancestral states. Syst Biol. 2001;50(3):351–366. 10.1080/10635150119871 12116580

[67] Ronquist F. Bayesian inference of character evolution. Trends Ecol Evol. 2004;19(9):475–481. 10.1016/j.tree.2004.07.002 16701310

[68] Vanderpoorten A, Goffinet B. Mapping uncertainty and phylogenetic uncertainty in ancestral character state reconstruction: An example in the moss genus *Brachytheciastrum*. Syst Biol. 2006;55(6):957–971. 10.1080/10635150601088995 17345677

[69] Litsios G, Salamin N. Effects of phylogenetic signal on ancestral state reconstruction. Syst Biol. 2012;61(3):533–538. 10.1093/sysbio/syr124 22223447

[70] Mandáková T, Lysak MA. Chromosomal phylogeny and karyotype evolution in *x* = 7 crucifer species (Brassicaceae). The Plant Cell. 2008;20(10):2559–2570. 10.1105/tpc.108.062166 18836039 PMC2590746

[71] Mandáková T, Joly S, Krzywinski M, Mummenhoff K, Lysak MA. Fast diploidization in close mesopolyploid relatives of *Arabidopsis*. The Plant Cell. 2010;22(7):2277–2290. 10.1105/tpc.110.074526 20639445 PMC2929090

[72] Hoffmann AA, Rieseberg LH. Revisiting the impact of inversions in evolution: From population genetic markers to drivers of adaptive shifts and speciation? Annu Rev Ecol Evol Syst. 2008;39:21–42. 10.1146/annurev.ecolsys.39.110707.173532 20419035 PMC2858385

[73] Kirkpatrick M. How and why chromosome inversions evolve. PLoS Biol. 2010;8(9):e1000501 10.1371/journal.pbio.1000501 20927412 PMC2946949

[74] Faria R, Navarro A. Chromosomal speciation revisited: rearranging theory with pieces of evidence. Trends Ecol Evol. 2010;25(11):660–669. 10.1016/j.tree.2010.07.008 20817305

[75] Pirie MD, Humphreys AM, Antonelli A, Galley C, Linder HP. Model uncertainty in ancestral area reconstruction: a parsimonious solution? Taxon. 2012;61(3):652–664.

[76] Nielsen R. Mapping mutations on phylogenies. Syst Biol. 2002;51(5):729–739. 10.1080/10635150290102393 12396587

[77] Huelsenbeck JP, Nielsen R, Bollback JP. Stochastic mapping of morphological characters. Syst Biol. 2003;52(2):131–158. 10.1080/10635150390192780 12746144

[78] Bollback JP. SIMMAP: Stochastic character mapping of discrete traits on phylogenies. BMC Bioinformatics. 2006;7:88 10.1186/1471-2105-7-88 16504105 PMC1403802

[79] Xiang QYJ, Thomas DT. Tracking character evolution and biogeographic history through time in Cornaceae—Does choice of methods matter? J Syst Evol. 2008;46(3):349–374.

[80] Grant V. Plant speciation. 2nd ed New York: Columbia University Press; 1981.

[81] Goldblatt P, Takei M. Chromosome cytology of Iridaceae, patterns of variation, determination of ancestral base numbers, and modes of karyotype change. Ann Mo Bot Gard. 1997;84:285–304. 10.2307/2400005

[82] Lysak MA, Berr A, Pecinka A, Schmidt R, McBreen K, Schubert I. Mechanisms of chromosome number reduction in *Arabidopsis thaliana* and related Brassicaceae species. Proc Natl Acad Sci USA. 2006;103(13):5224–5229. 10.1073/pnas.0510791103 16549785 PMC1458822

[83] Bakker FT, Culham A, Pankhurst CE, Gibby M. Mitochondrial and chloroplast DNA-based phylogeny of *Pelargonium* (Geraniaceae). Am J Bot. 2000;87(5):727–734. 10.2307/2656859 10811797

[84] Ayala FJ, Coluzzi M. Chromosome speciation: humans, Drosophila, and mosquitoes. Proc Natl Acad Sci USA. 2005;102 Suppl. 1:6535–6542. 10.1073/pnas.0501847102 15851677 PMC1131864

