# Supplementary material for: The Impact of Reconstruction Methods, Phylogenetic Uncertainty and Branch Lengths on Inference of Chromosome Number Evolution in American Daisies (Melampodium, Asteraceae)
Source: PLoS One. 2016 Sep 9;11(9):e0162299. doi: 10.1371/journal.pone.0162299 (PMC5017664; doi:10.1371/journal.pone.0162299)
Supplement: S1 Fig — G-L distributions reconstructed using maximum likelihood in ChromEvol (ML-CE, grey) and in BayesTraits (ML-BT, shades of red) on phylogenetic trees obtained from analyses of (A) nuclear sequence data using BEAST (ITS-B), (B) nuclear sequence data using MrBayes (ITS-MB), (C) plastid sequence data using BEAST (matK-B) and (D) plastid sequence data using MrBayes (matK-MB) before (dark grey and orange) and after (light grey and red) pruning Melampodium repens, the sole species with n = 14, and restricting minimum and maximum chromosome number to 9 and 12, respectively. (PDF) [file pone.0162299.s001.pdf]

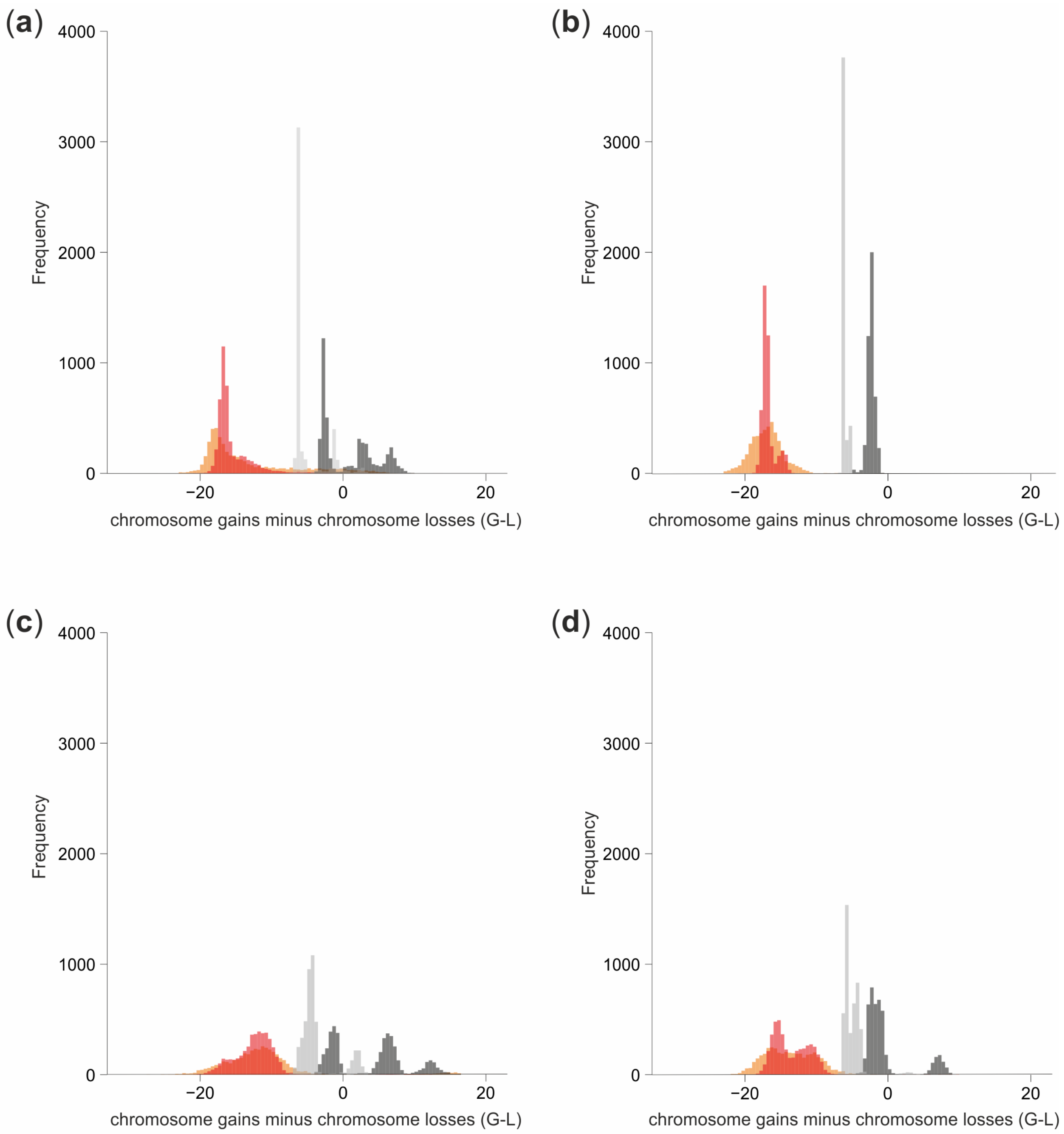

S1 Fig

Distributions of the number of chromosome gains minus the number of chromosome losses (G-L) with and without *Melampodium repens*. G-L distributions reconstructed using maximum likelihood in ChromEvol (ML-CE, grey) and in BayesTraits (ML-BT, shades of red) on phylogenetic trees obtained from analyses of (a) nuclear sequence data using BEAST (ITS-B), (b) nuclear sequence data using MrBayes (ITS-MB), (c) plastid sequence data using BEAST (matK-B) and (d) plastid sequence data using MrBayes (matK-MB) before (dark grey and orange) and after (light grey and red) pruning *Melampodium repens*, the sole species with $n = 14$, and restricting minimum and maximum chromosome number to 9 and 12, respectively.
