# Supplementary material for: The Impact of Reconstruction Methods, Phylogenetic Uncertainty and Branch Lengths on Inference of Chromosome Number Evolution in American Daisies (Melampodium, Asteraceae)
Source: PLoS One. 2016 Sep 9;11(9):e0162299. doi: 10.1371/journal.pone.0162299 (PMC5017664; doi:10.1371/journal.pone.0162299)
Supplement: S2 Fig — Relationship between root state and G-L reconstructed using maximum likelihood in ChromEvol (ML-CE) on phylogenetic trees obtained from analyses of (A) nuclear sequence data using BEAST (ITS-B), (B) nuclear sequence data using MrBayes (ITS-MB), (C) plastid sequence data using BEAST (matK-B) and (D) plastid sequence data using MrBayes (matK-MB). (PDF) [file pone.0162299.s002.pdf]

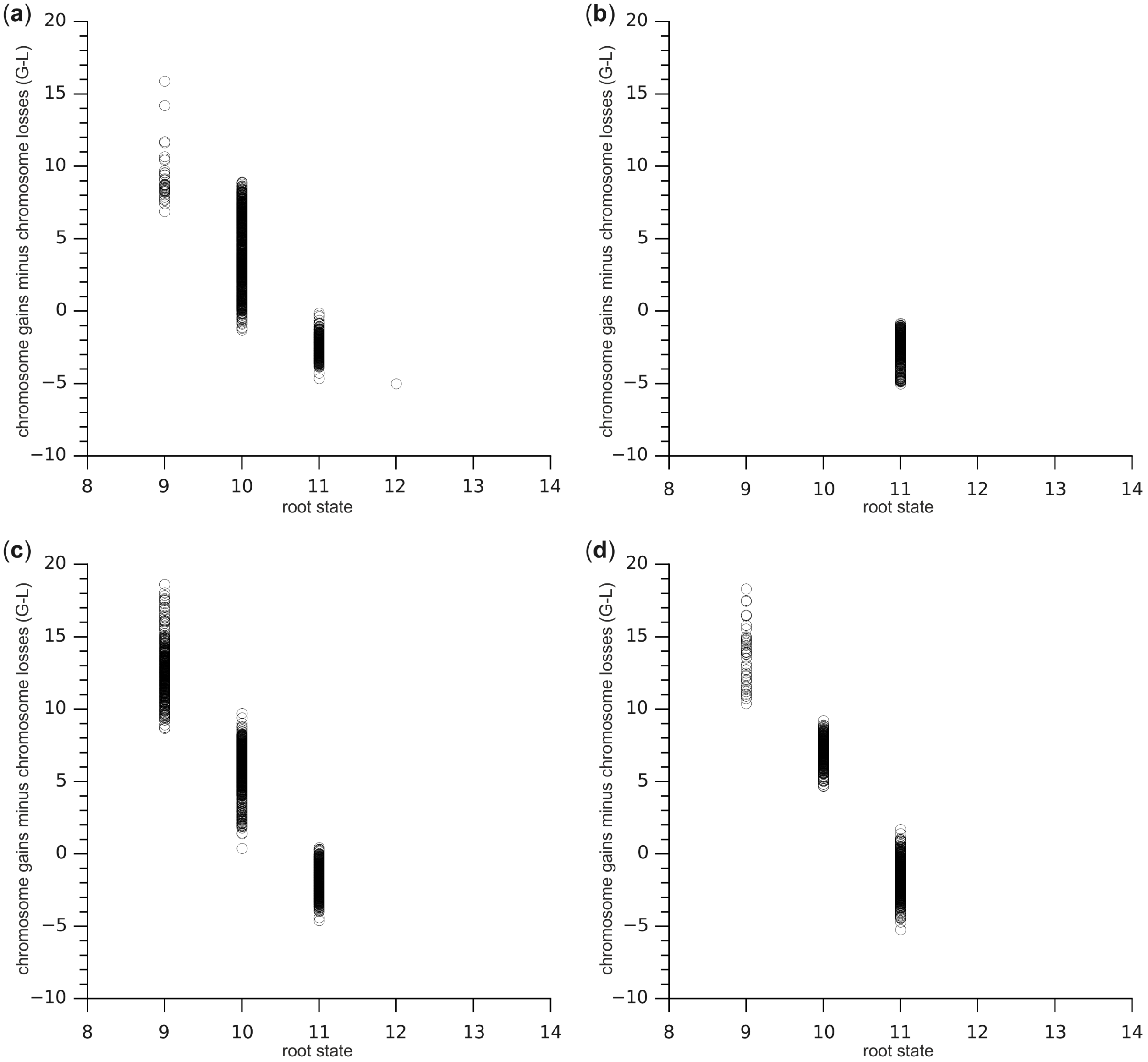

S2 Fig

Relationship between root state and the number of chromosome gains minus the number of chromosome losses (G-L). Relationship between root state (x-axis) and G-L (y-axis) reconstructed using maximum likelihood in ChromEvol (ML-CE) on phylogenetic trees obtained from analyses of (a) nuclear sequence data using BEAST (ITS-B), (b) nuclear sequence data using MrBayes (ITS-MB), (c) plastid sequence data using BEAST (matK-B) and (d) plastid sequence data using MrBayes (matK-MB).
