# Supplementary material for: The Impact of Reconstruction Methods, Phylogenetic Uncertainty and Branch Lengths on Inference of Chromosome Number Evolution in American Daisies (Melampodium, Asteraceae)
Source: PLoS One. 2016 Sep 9;11(9):e0162299. doi: 10.1371/journal.pone.0162299 (PMC5017664; doi:10.1371/journal.pone.0162299)
Supplement: S3 Fig — G-L distributions reconstructed using maximum likelihood in ChromEvol (ML-CE) on phylogenetic trees obtained from analyses of (A) nuclear sequence data using MrBayes (ITS-MB) and (B) plastid sequence data using MrBayes (matK-MB) before (black) and after (grey) ultrametricization using PATHd8. (PDF) [file pone.0162299.s003.pdf]

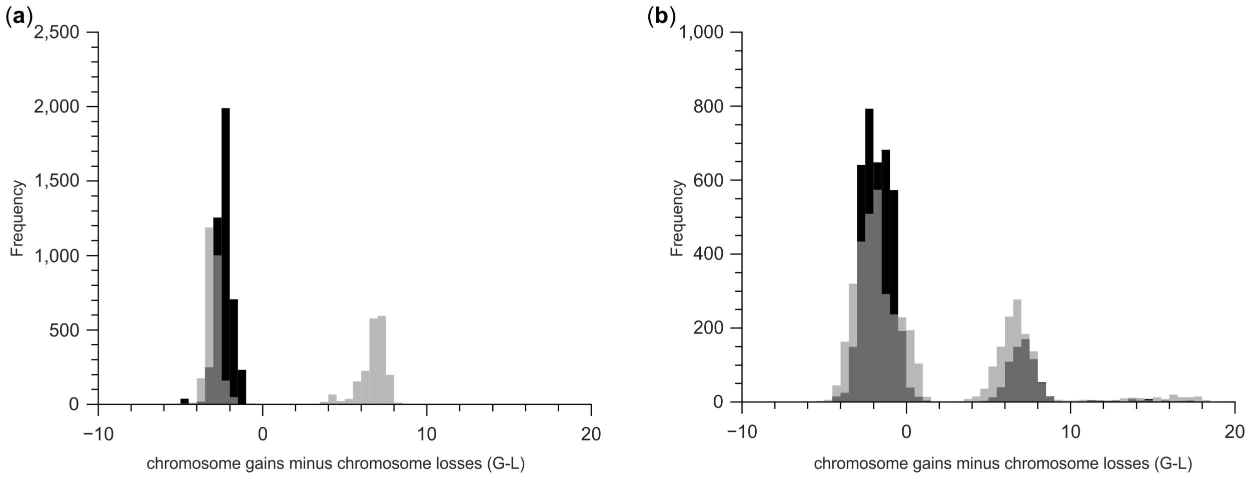

S3 Fig

Distributions of the number of chromosome gains minus the number of chromosome losses (G-L) before and after ultrametricization. G-L distributions reconstructed using maximum likelihood in ChromEvol (ML-CE) on phylogenetic trees obtained from analyses of (a) nuclear sequence data using MrBayes (ITS-MB) and (b) plastid sequence data using MrBayes (matK-MB) before (black) and after (grey) ultrametricization using PATHd8.
